# Gene Flow and Hybridization between Numerically Imbalanced Populations of Two Duck Species in the Falkland Islands

**DOI:** 10.1371/journal.pone.0023173

**Published:** 2011-08-26

**Authors:** Kevin G. McCracken, Robert E. Wilson

**Affiliations:** Institute of Arctic Biology, University of Alaska Museum, and Department of Biology and Wildlife, University of Alaska Fairbanks, Fairbanks, Alaska, United States of America; Ecole Normale Supérieure de Lyon, France

## Abstract

Interspecific hybridization is common in plants and animals, particularly in waterfowl (Anatidae). One factor shown to contribute to hybridization is restricted mate choice, which can occur when two species occur in sympatry but one is rare. The Hubbs principle, or “desperation hypothesis,” states that under such circumstances the rarer species is more likely to mate with heterospecifics. Here we report interspecific hybridization between two waterfowl species that coexist in broad sympatry and mixed flocks throughout southern South America. Speckled teal (*Anas flavirostris*) and yellow-billed pintails (*Anas georgica*) are abundant in continental South America, but in the Falkland Islands speckled teal outnumber yellow-billed pintails approximately ten to one. Using eight genetic loci (mtDNA and 7 nuclear introns) coupled with Bayesian assignment tests and relatedness analysis, we identified a speckled teal x yellow-billed pintail F_1_ hybrid female and her duckling sired by a male speckled teal. Although our sample in the Falkland Islands was small, we failed to identify unequivocal evidence of hybridization or introgression in a much larger sample from Argentina using a three-population “isolation with migration” coalescent analysis. While additional data are needed to determine if this event in the Falkland Islands was a rare singular occurrence, our results provide further support for the “desperation hypothesis,” which states that scarcity in one population and abundance of another will often lead to hybridization.

## Introduction

Interspecific hybridization is an important mechanism of lineage diversification and adaptation in plants [1], [2], [3], and it has also been shown to be an important evolutionary force in animals [4], [5], [6]. Birds are no exception; at least one in ten species is known to hybridize [7], [8], [9], [10]. The waterfowl (Anatidae) comprise more than half of known avian hybrids [11], [12], [13]. Numerous factors have been implicated in the ability of the Anatidae to hybridize [12], [14], including Haldane's [15] rule. One factor in particular is that hybridization is encouraged by restricted mate choice, and is therefore common in areas where two species occur in sympatry but one species is rare [14]. This concept was first formalized by Hubbs [16]: “Great scarcity of one species coupled with the abundance of another often leads to hybridization: the individuals of the sparse species seem to have difficulty in finding their proper mates.” Hubbs referred to this principle as the “desperation hypothesis,” for which empirical support has now been found among numerous species of birds, including waterfowl [14].

Here, using multi-locus genetic data, we report an example of interspecific hybridization between two waterfowl species that exist in widespread sympatry throughout southern South America, but which show hybridization in a numerically imbalanced situation on the Falkland Islands. Both species, speckled teal (*Anas flavirostris*) and yellow-billed pintails (*Anas georgica*) are common throughout continental South America, but in the Falkland Islands one species is common and the other is rare [17], [18], [19]. Speckled teal are estimated to number approximately 6,000–11,000 breeding pairs in the Falkland Islands, whereas yellow-billed pintail breeding pairs likely number 600–1,000 [20]. The order-of-magnitude numerical imbalance in speckled teal and yellow-billed pintail population sizes in the Falkland Islands thus stands in contrast to continental South America, where each species is common and populations likely exceed 1,000,000 individuals.

Using data from eight genetic loci and Bayesian assignment tests and coalescent models, we identified an F_1_ female hybrid and her duckling in a small sample of 15 speckled teal banded in the Falkland Islands, but we found no evidence of hybridization or introgression among 56 speckled teal and 64 yellow-billed pintails collected over an area of sympatry in southern Argentina. Our results provide further support for Hubbs's [16] principle, the “desperation hypothesis.” Our study also revealed significant haplotype and allele frequency differences between speckled teal populations in the Falkland Islands and Argentina, suggesting that gene flow is restricted.

## Materials and Methods

### Specimen Collection

**Figure 1:**
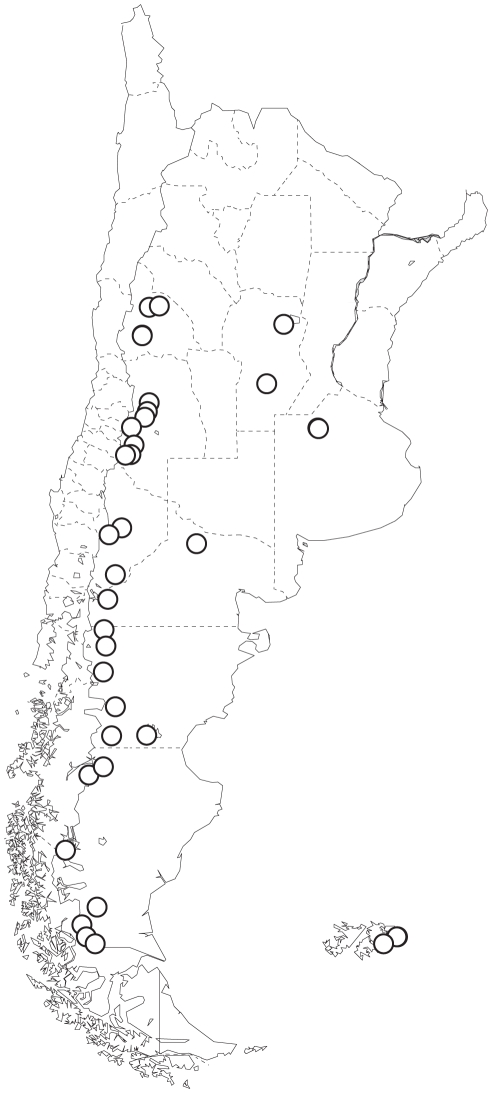
Localities of 71 speckled teal sampled in Argentina and the Falkland Islands.

Speckled teal (*n* = 56) and yellow-billed pintails (*n* = 64) were collected at widespread localities in Argentina between 2001 and 2005, and blood samples were obtained from speckled teal banded on East Falkland Island (*n* = 15) in 2002. Localities of speckled teal are illustrated in Figure 1. Localities for yellow-billed pintails are illustrated in figure 1 of McCracken et al. [21]. All specimens in Argentina were collected at elevations <2,100 meters, and all of the speckled teal in our study were identified as the nominate subspecies *Anas f. flavirostris*. Vertebrate collecting activities were approved by the University of Alaska Fairbanks Institutional Animal Care and Use Committee (IACUC 02-01, 05-05) and by federal and provincial governments in Argentina and the Falkland Islands (D.F.S. No. 3209/01, 13168/03, 13169/03, 20419/05, 20420/05).

**Figure 2:**
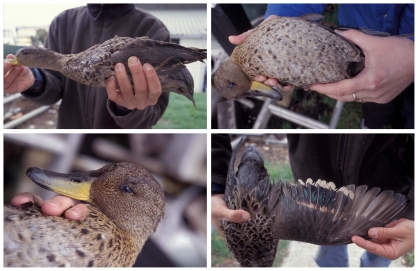
F_1_ speckled teal x yellow-billed pintail hybrid (REW 325).

One adult female from the Falkland Islands (REW 325) and her duckling (REW 324) were identified in the field as possible hybrids. An unsampled male speckled teal was observed tending the brood with the female. Yellow-billed pintails are larger than speckled teal, and REW 325 was more similar to yellow-billed pintail in wing chord, culmen, and tarsus measurements. Along with body size, the female had some plumage characteristics typical of a yellow-billed pintail (Fig. 2). The back was uniform brown with large, dark scales, and the brown head feathers seemed more like pintail feathers. However, the speculum was bright green as found in speckled teal, but extended further out on the secondaries like yellow-billed pintail. The body, head, and neck shapes were more similar to a speckled teal, which is stockier than the more slender appearance of the yellow-billed pintail.

### DNA Extraction, PCR, and Sequencing

DNA was isolated from frozen muscle or blood using standard protocols with DNeasy Tissue Kits (QIAGEN, Valencia, California). Eight gene regions including the mitochondrial DNA (mtDNA) control region and seven nuclear loci were sequenced (Table 1). Methods describing PCR and DNA sequencing protocols are described in McCracken et al. [21]. Sequences and specimen voucher information, including geo-referenced localities, are available in GenBank (accession numbers FJ617587–FJ617592, FJ617597–FJ617598, FJ617634–FJ617670, FJ617677–FJ617694, FJ617702, FJ617703–FJ617708, FJ617713–FJ617714, FJ617750–FJ617784, FJ617791–FJ617808, FJ617816, FJ617817–FJ617822, FJ617827–FJ617828, FJ617864–FJ617900, FJ617907–FJ617924, FJ617932, FJ617933–FJ617938, FJ617943–FJ617944, FJ617980–FJ618016, FJ618023–FJ618040, FJ618048, FJ618049–FJ618054, FJ618059–FJ618060, FJ618096–FJ618132, FJ618139–FJ618156, FJ618164, FJ618165–FJ618170, FJ618175–FJ618176, FJ618212–FJ618248, FJ618255–FJ618272, FJ618280, FJ618281–FJ618286, FJ618291–FJ618292, FJ618328–FJ618364, FJ618371–FJ618388, FJ618396, FJ618397–FJ618402, FJ618407–FJ618408, FJ618444–FJ618480, FJ618487–FJ618504, FJ618512, GQ269874–GQ269943, GQ270014–GQ270084, GQ270155–GQ270225, GQ270296–GQ270372, GQ270476–GQ270546, GQ271325–GQ271395, GQ272063–GQ272132, and JN223305–JN223375).

**Table 1:** Genes sequenced and their chromosomal positions in the chicken genome.

Locus	Base pairs sequenced	Chicken chromosome
mtDNA control region (mtDNA)	976–981	mtDNA
Ornithine decarboxylase intron 5 (ODC1)	352	3
*α* enolase intron 8 (ENO1)	314	21
*β* fibrinogen intron 7 (FGB)	246	4
N-methyl D aspartate 1 glutamate receptor intron 11 (GRIN1)	328–744	17
Phosphoenolpyruvate carboxykinase intron 9 (PCK1)	345–351	20
*α*A hemoglobin subunit (HBA2)	677–678	14
*β*A hemoglobin subunit (HBB)	1,576–1,582	1

Location in the chicken genome as defined by Hillier et al. [68].

### Allelic Phase Determination

The allelic phase of each nuclear sequence that was heterozygous at two or more nucelotide positions was determined independently for each species using allele-specific priming and the software PHASE 2.1 [22]. PHASE uses a Bayesian method to infer haplotypes from diploid genotypic data while incorporating recombination and the decay of linkage disequilibrium (LD) with distance. We first analyzed each composite sequence of both alleles using the default software settings (100 main iterations, 1 thinning interval, 100 burn-in) followed by 1,000 main iterations and 1,000 burn-in (−×10 option) for the final iteration. The PHASE algorithm was run five times (−×5 option) from different starting points, selecting the result with the best overall goodness of fit. For individuals with allele pair probabilities <80%, we then designed allele-specific primers to selectively amplify a single allele [23], [24]. The resulting haploid allele sequence was then subtracted from the diploid composite sequence to obtain the gametic phase of the second allele. Each data set was then analyzed five more times using PHASE and the additional known allele sequences (−k option). PHASE analyses and allele specific priming were performed for the complete sequences of the ODC1, ENO1, FGB, GRIN1, and PCK1 introns and the HBA2 gene. For the HBB gene, which was shown to exhibit high levels of recombination in speckled teal [25], we analyzed a subset of the 3′ HBB sequence consisting of the last 291 bp of intron two and exon three. The gametic phase of each autosomal sequence was identified experimentally or with >95% posterior probability for approximately 95% of the inviduals included in each data set.

### Analysis of Genetic Differentiation between and within Species

To characterize genetic differentiation between speckled teal and yellow-billed pintails and examine how these patterns varied among loci, we calculated *Φ_ST_* between species using the software Arlequin 3.5 [26] and illustrated networks for each locus using the median-joining algorithm in the software NETWORK 4.6 ([27]; Fluxus Technology, Ltd.). Additionally, we calculated *Φ_ST_* between the Falkland Islands and Argentina for speckled teal, as well as other measures of genetic diversity such as the total number of polymorphic positions, frequency of the most common allele in each population, and nucleotide diversity (*π*/site). Because speckled teal sampling was unbalanced between the island and mainland sites, allelic richness was standardized to the smallest sample size (30 alleles for autosomal loci and 15 haplotypes for mtDNA).

### Identification of Admixed Individuals

We used the software Structure 2.2 [28] to identify speckled teal and yellow-billed pintail individuals with admixed ancestry and compute their probability of assignment to respective populations. The eight-locus sequence data were first converted to numerical genotypic data using the software Collapse [29], with missing data coded in place of a second allele for the mtDNA. In the first step of the analysis, a simple two-population model (*K* = 2) was conducted using the admixture model (*α* = 1) with independent allele frequencies (*λ* = 1) and no *a priori* population information (POPFLAG = 0). In the second step, individuals with assignment probabilities >0.99 as determined in the first analysis were pre-assigned to their respective clusters corresponding to populations 1 and 2 (POPFLAG = 1). The ancestry of individuals with assignment probabilities <0.99 in the first analysis was then re-estimated (POPFLAG = 0) using allele frequencies defined by individuals previously determined to have posterior probabilities >0.99. Information about the allele frequencies from pre-defined individuals was thus used to improve the accuracy of inference about the admixture of unknown individuals. We used RE-RAT [30] to estimate Queller and Goodnight's [31] pairwise, symmetric relatedness statistic (*r_xy_*) and compute the average relatedness for each population.

We also used Structure's two-population model (*K* = 2) to compute the probability of assignment for speckled teal individuals inhabiting the Falkland Islands and Argentina (yellow-billed pintails excluded). No prior population information was incorporated into this analysis, and the same run parameters described above were used, with the analysis repeated five times.

### Isolation with Migration Analysis

We further assessed evidence for hybridization between speckled-teal and yellow-billed pintails using “Isolation with Migration” in IMa2 [32], which allows for analysis of divergence and gene flow between two or more populations. In this case, we estimated the effective population size parameters (*θ*), time since divergence (*t*), and gene flow rates (*M*) in both directions between two populations of speckled teal and one population of yellow-billed pintails (Table 2).

**Table 2:** Estimated parameters in the three-population IMa2 analysis.

Parameter	Symbol	Population/divergence/gene flow
Population size parameter (4*N_e_μ*)	*θ_AR_*	Speckled teal in Argentina
	*θ_FK_*	Speckled teal in Falkland Islands
	*θ_YP_*	Yellow-billed pintail
	*θ_0_*	*θ* ancestral at *t_0_*
	*θ_1_*	*θ* ancestral at *t_1_*
Time since divergence (*t*)	*t_0_*	Between Argentine speckled teal and Falkland Islands speckled teal
	*t_1_*	Between speckled teal and yellow-billed pintail
Gene flow (*m/μ*)	*M_AR>FK_*	Into Argentine speckled teal from Falkland Islands speckled teal
	*M_FK>AR_*	Into Falkland Islands speckled teal from Argentine speckled teal
	*M_AR>YP_*	Into Argentine speckled teal from yellow-billed pintails
	*M_YP>AR_*	Into yellow-billed pintails from Argentine speckled teal
	*M_FK>YP_*	Into Falkland Islands speckled teal from yellow-billed pintails
	*M_YP>FK_*	Into yellow-billed pintails from Falkland Islands speckled teal

Because the IM model assumes that all sequences are free from intra-locus recombination, we tested for recombination using the four-gamete test [33] implemented in DNAsp 4.10 [34]. Evidence of recombination was detected for all nuclear loci except FGB. The other loci were therefore truncated to include the longest fragment with no apparent recombination. For ODC1 this included positions 1–151, ENO1 positions 1–172, GRIN1 positions 75–178, PCK1 positions 1–254, and HBA2 positions 412–678. HBB was not included in the analysis as few segregating sites were retained after removing recombining blocks of sequence. The mtDNA was omitted because it was highly divergent and reciprocally monophyletic between speckled teal and yellow-billed pintails; no mtDNA haplotypes were shared between species (see below). A total of six nuclear loci were thus included in the IMa2 analysis. The HKY [35] substitution model was used in the IMa2 analysis, as opposed to the infinite-sites model, because all six loci possessed three or more alleles at one or more sites.

IMa2 was first run with wide priors to explore the sensitivity of parameter estimates to different upper bounds. The analyses were then conducted with uniform priors that encompassed the full posterior distribution of each parameter from the preliminary runs (*θ* = 5, *t* = 2, and *M* = 100). The upper bound for *t* was selected based on the assumption that time since divergence could not exceed TMRCA (time to most recent common ancestor of all sequences). The Markov chain Monte Carlo was run for 15 million steps, sampling the posterior distribution every 50 steps for a total of 300,000 sampled genealogies, with a burn-in of 500,000 steps. All runs included 20 chains with a geometric heating scheme. Autocorrelation was monitored during the run, and analyses were repeated five times with different random number seeds to ensure that parameter estimates converged.

Parameter estimates for *θ* and *t* were converted to biologically meaningful values using published estimates of generation time and the geometric mean of the substitution rate (*μ* per locus) calculated for the same six loci in *Anas* ducks using fossil dates calibrated to the duck/snow goose (*Anser caerulescens*) split (see McCracken et al. [21], [25]). The number of effective migrant individuals per generation was obtained by multiplying *θ* (4*N_e_μ*) by *M* (*m/μ*) to obtain 4*N_e_m*.

To determine whether yellow-billed pintail alleles had introgressed into speckled teal or vice versa, we examined the resulting posterior distribution for the four pertinent gene flow estimates shown in Table 2. Estimates of *M_AR>YP_*, *M_YP>_*_AR_, *M*_FK*>*YP_, or *M*_YP*>FK*_ (see Table 2 for definitions) with a lower 95% confidence interval that did not overlap zero were interpreted as quantitatively strong evidence for hybridization, whereas estimates of *M* that overlapped zero could not be decisively interpreted as evidence of gene flow. Finally, the timing of each inferred interspecific gene flow event was recorded for all loci in each sampled genealogy, and the posterior distribution of timing of these events was compared to the posterior distribution of *t* between the speckled teal and pintail lineages.

## Results

### Genetic Differentiation between Speckled Teal and Yellow-billed Pintails

**Figure 3:**
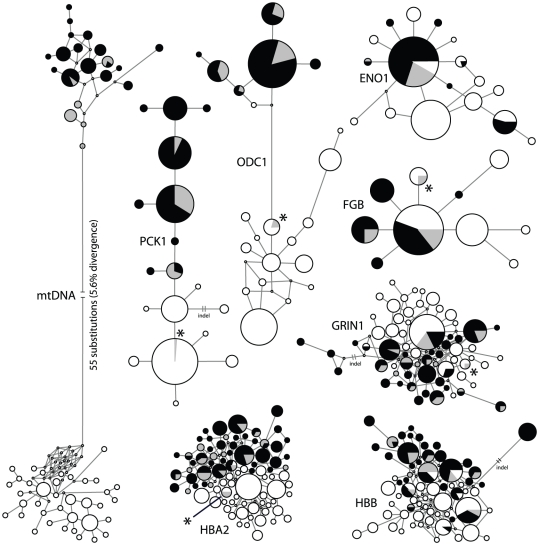
Networks for eight genetic loci. Speckled teal alleles are illustrated in black (Argentina) and grey (Falkland Islands), and yellow-billed pintail alleles are illustrated in white. Circle area is proportional to the number of shared alleles. Asterisks indicate the positions of putatively introgressed yellow-billed pintail alleles for two individuals (REW 325 and REW 325) in the Falkland Islands.

Speckled teal and yellow-billed pintails were significantly differentiated at all loci, with *Φ_ST_* values ranging from 0.11 to 0.94 (Table 3). MtDNA haplotype groups were reciprocally monophyletic (*Φ_ST_* = 0.94), and uncorrected divergence between species clusters was 5.6% (Fig. 3). The two aforementioned individuals (REW 325, 324) possessed speckled teal mtDNA haplotypes, not yellow-billed pintail haplotypes. ODC1 (*Φ_ST_* = 0.81) was quasi-reciprocally monophyletic; two apparent species clusters were separated by 2.6% uncorrected sequence divergence. One yellow-billed pintail (KGM 750) that was morphologically indistinguishable from other yellow-billed pintails possessed a private singleton ODC1 allele that was one to three bases divergent from three other alleles in the speckled teal cluster, and REW 325 and 324 each possessed one identical allele that was shared with eight yellow-billed pintails, but not speckled teal. PCK1 (*Φ_ST_* = 0.77) possessed no shared alleles, except for REW 325, which possessed the most common allele found in yellow-billed pintail on one chromosome and an allele shared by speckled teal on the other chromosome. Among the other loci, ENO1 and FGB had low allelic diversity and two and three alleles shared between species, respectively, whereas GRIN1, HBA2, and HBB had higher allelic diversity and two to six shared alleles. In total, REW 325 had five alleles at five loci that were shared exclusively with yellow-billed pintails, and REW 324 had four such alleles at three loci.

**Table 3:** Patterns of allele sharing and *Φ_ST_* between speckled teal and yellow-billed pintails.

Locus	No. alleles (speckled teal/yellow-billed pintail)	No alleles shared between species	*Φ_ST_*
mtDNA control region	21/37	0	0.94
Ornithine decarboxylase	7/16	1 (1)	0.81
*α* enolase	7/13	3 (0)	0.34
*β* fibrinogen	6/6	2 (1)	0.20
N-methyl D aspartate 1 glutamate receptor	35/34	3 (1)	0.11
Phosphoenolpyruvate carboxykinase	10/9	1 (1)	0.77
*α*A hemoglobin	48/40	2 (1)	0.28
*β*A hemoglobin	32/39	6 (0)	0.15

All *Φ_ST_* values were significant (P<0.000001). Tamura-Nei [69] substitution model was used to calculate *Φ_ST_*. Number in parentheses indicates the number of speckled teal alleles from Falkland Islands shared exclusively with yellow-billed pintails (and not speckled teal from Argentina).

### Identification of Admixed Individuals

In the Structure analysis with no prior population information, one yellow-billed pintail (KGM 1250) that was morphologically indistinguishable from other yellow-billed pintails was assigned to population cluster 1 with <0.99% posterior probability (*P* = 0.978). Two speckled teal (REW 325 and 324) were assigned to cluster 2 with <0.99% posterior probability (*P* = 0.812 and 0.978, respectively). In the second analysis in which prior assignments were used to preassign all but these three individuals, KGM 1250 was assigned to the yellow-billed pintail population with *P* = 0.961, and REW 325 and 324 were assigned to the speckled teal population with *P* = 0.630 and *P* = 0.860, respectively. The KGM 1250 individual was thus assigned to the yellow-billed pintail cluster with high probability, whereas REW 325 and REW 324 exhibited admixed ancestry.

REW 325 and 324 shared the same mtDNA haplotype as expected and one allele each at five of seven nuclear loci. Both pairs of alleles were identical at the other two loci. Based on this finding and the above referenced results, REW 325 was likely an F_1_ hybrid sired by a male yellow-billed pintail and female speckled teal that then successfully backcrossed to a male speckled teal and produced the REW 324 duckling. Queller and Goodnight [31] relatedness (*r_xy_*) for REW 325 and 324 was 0.564. Average relatedness for the speckled teal population as a whole was 0.009±0.004 (SE) and 0.013±0.002 (SE) for the yellow-billed pintail population.

### Gene Flow between Speckled Teal and Yellow-billed Pintails

The three-population IMa2 coalescent analysis corroborated the results of the assignment tests. In proceeding further, it is useful to note that all estimates of the gene flow rate parameter *M* in coalescent genealogy samplers like IM are scaled to the mutation rate (*M* = *m*/*μ*), so *M* is thus the ratio of gene flow to mutation. The number of effective immigrants (4*N_e_m*) is obtained by multiplying the scaled gene flow rate *M* by *θ*. Confidence intervals are reported for the 95% highest posterior density (HPD).

**Figure 4:**
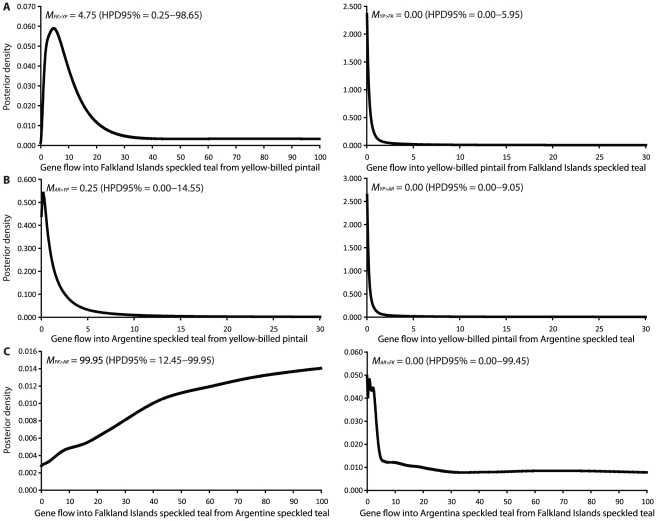
Gene flow (*M* = *m*/*μ*) estimates from the IMa2 analysis of six nuclear loci.

**Figure 5:**
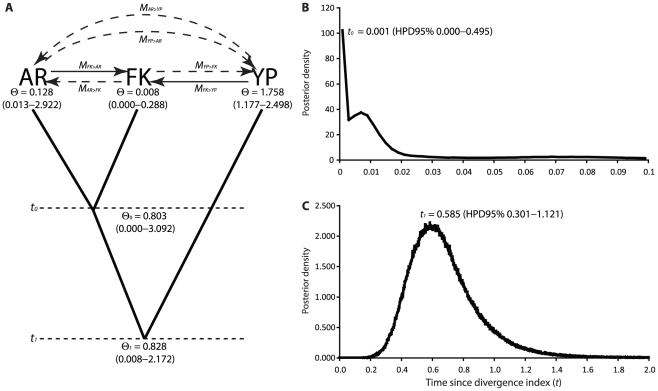
Three-population IMa2 model for speckled teal (AR = Argentina, FK = Falkland Islands) and yellow-billed pintails (YP) for six nuclear loci. A) Effective population size parameter *θ*. B) Time since divergence index (*t*) between speckled teal in the Falkland Islands and Argentina. C) Time since divergence index (*t*) between speckled teal and yellow-billed pintails. The 95% highest posterior densities (HPD) for each parameter are shown in parentheses (see Table 2 for definitions). Arrows depict the six gene flow parameters (*M* = *m*/*μ*). HPD95% estimates of *M* that overlap zero are shown with dashed lines (as shown in Figure 4).

Gene flow from yellow-billed pintails into the Falkland Islands speckled teal population was statistically greater than zero (*M_FK>YP_* = 4.75, HPD95% = 0.25–98.65; Fig. 4A), suggesting that yellow-billed pintails have introduced new mutations into the Falkland Islands specked teal population at rate equivalent to approximately five times the substitution rate. Furthermore, the timing of inferred gene flow events (*t*) between Falkland Islands speckled teal and pintails peaked sharply at zero time before present (HPD95% = 0.000–0.049). Interspecific gene flow therefore postdated the confidence intervals for the timing of divergence (*t*) between the speckled teal and pintail lineages (Fig. 5) by a wide margin. Identical alleles therefore could not be attributed to ancient coalescence of ancestral polymorphisms but could only be explained by recent gene flow. By contrast, gene flow between yellow-billed pintails and speckled teal in Argentina could not be distinguished from zero (*M_AR>YP_* = 0.25, HPD95% = 0.00–14.55; Fig. 4B), and has likely occurred at a rate lower than the substitution rate. It is difficult to estimate the total number of yellow-billed pintails hybridizing with speckled teal in the Falkland Islands from this type of data because variance in *M* and *θ* must be considered jointly. Multiplying the point estimate of *M_FK>YP_* (4.75) by the point estimate of *θ_FK_* (0.0075) suggests that the number of effective yellow-billed pintail immigrants is less than one per generation. But if the upper 95% HPD of both *M* (98.65) and *θ* (0.2875) are considered, the number could be as high as 28 immigrants per generation.

Gene flow between speckled teal in Argentina and the Falklands Islands was asymmetric. Gene flow from Argentina to the Falkland Islands (*M_FK>AR_*) peaked at the upper prior (*M* = 100), so the true value is likely higher (Fig. 4C). Gene flow in the opposite direction, from the Falkland Islands to Argentina could not be distinguished from zero, but could just as well be greater as the tail did not asymptotically approach the x-axis (*M_AR>FK_* = 0.05, HPD95% = 0.00–99.45). The posterior density distributions for *M_FK>AR_* and *M_AR>FK_* were not smooth, which is a common outcome in IM when low *Φ_ST_* values are observed for most loci (see below).

Given equal substitution rates among populations, which is likely a valid assumption for closely related species, the effective population size (*N_e_*) of the Argentine population was 17 times greater than the Falkland Islands, and *N_e_* for yellow-billed pintails was more than ten times greater than speckled teal (Fig. 5A). Time since divergence between the Argentine and Falklands Islands populations of speckled teal is likely very recent, the posterior probability of *t* peaked sharply at zero (*t* = 0.001, HPD95% = 0.00–0.495; Fig. 5B). The peak was bimodal, however, suggesting that divergence could be older (*t* = 0.007). By contrast, divergence between speckled teal and yellow-billed pintails was much deeper (*t* = 0.585, HPD95% = 0.301–1.121; Fig. 5C). Based on a point estimate substitution rate of 3.93×10^−7^ substitutions/locus/year obtained for the same six loci from five species of *Anas* ducks [25], divergence between speckled teal and yellow-billed pintails might date between approximately 0.77 and 2.85 million years.

### Genetic Differentiation between the Falkland Islands and Argentina

**Figure 6:**
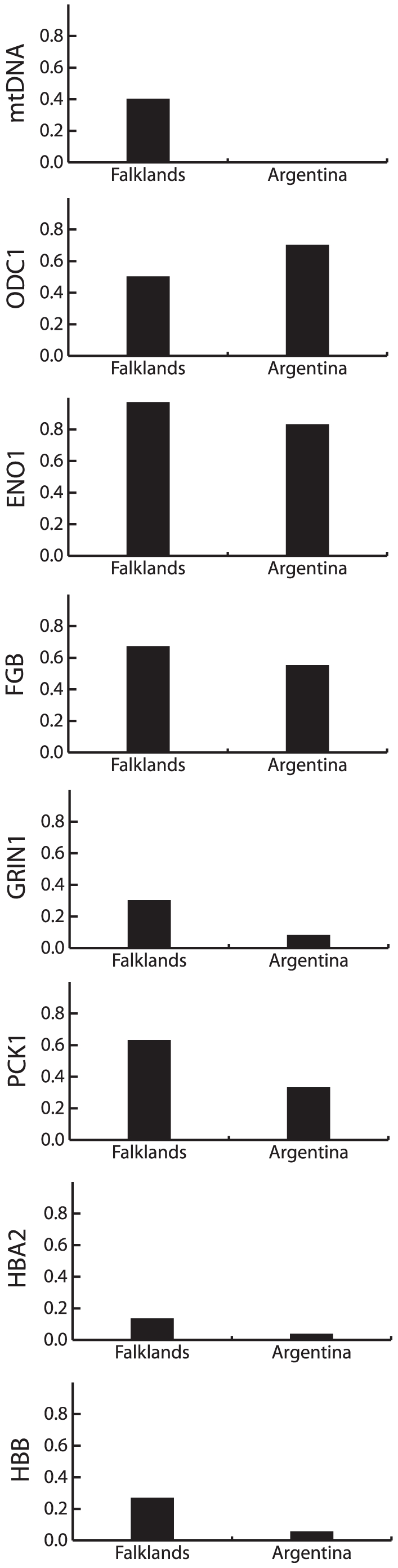
Frequency of the most common speckled teal allele in the Falkland Islands and Argentina.

Speckled teal exhibited highly significant mtDNA differentiation between the Falkland Islands and Argentina (*Φ_ST_* = 0.45; Table 4). Sixteen mtDNA haplotypes were found in Argentina, whereas six haplotypes were sampled in the Falkland Islands (Fig. 3). Two haplotypes were shared between the Falkland Islands and Argentina, and the other four, including the most common haplotype in the Falklands, were not observed in Argentina (Fig. 6, Table 4). MtDNA allelic richness was greater in Argentina, but nucleotide diversity (*π*/site) was greater in the Falklands.

**Table 4:** Genetic diversity measures (Argentina/Falkland Islands) and *Φ_ST_* between mainland and island populations of speckled teal.

Locus	Variable sites	Alleles	Standardized allelic richness in Argentina (±SD)	Nucleotide diversity (*π*/site)	Private alleles in the Falkland Islands	*Φ_ST_*
mtDNA control region	20/10	16/6	9±1	0.002121/0.004014	4	**0.42**
Ornithine decarboxylase	5/12	6/5	5±1	0.002177/0.006800	0	**0.05**
*α* enolase	7/1	7/2	4±1	0.002358/0.000217	0	0.05
*β* fibrinogen	4/2	5/3	4±4	0.003074/0.002284	0	0.05
N-methyl D aspartate 1 glutamate receptor	28/13	31/14	16±2	0.009604/0.007949	3	0.00
Phosphoenolpyruvate carboxykinase	7/5	9/4	6±1	0.003535/0.003295	0	**0.23**
*α*A hemoglobin	20/15	39/19	19±2	0.005497/0.005012	8	**0.02**
*β*A hemoglobin	21/11	31/10	16±2	0.012179/0.013172	1	0.01

*Φ_ST_* values in bold text were significant (*P*<0.05). Tamura-Nei [69] substitution model was used to calculate *Φ_ST_*.

In contrast to the mtDNA, most nuclear loci showed less population differentiation. Three loci (ODC1, PCK1, and HBA2) yielded significant *Φ_ST_* values ranging from 0.02 to 0.23 (*P*<0.05; Table 4). Eight private alleles were observed for HBA2 in the Falklands, and three and one private alleles were found in GRIN1 and HBB, respectively. In all but one case (ODC1), the most common allele occurred in Argentina at lower frequency than in the Falklands (Fig. 6). Nuclear allelic richness either did not differ or was greater in Argentina.

For speckled teal, the average assignment probabilities (±SD) to the Falkland Islands and Argentina were 0.59±0.04 and 0.55±0.03, respectively. Despite strong differentiation in the mtDNA, low differentiation and evidence of high gene flow in the nuclear DNA resulted in little power to discriminate between island and continental populations of speckled teal.

## Discussion

Interspecific hybridization is not uncommon in birds, especially the waterfowl. Indeed, the majority of known avian hybrids are represented by the Anatidae [13], and hybridization has been studied previously in a variety of waterfowl species using genetic assays [36], [37], [38], [39], [40], [41], [42]. Key factors contributing to hybridization in the waterfowl have been shown to include a tendency for hybridization to occur among more closely related species and between species that coexist in sympatry [12]. Randler [14], likewise, found that hybridization occurs more frequently when one species is common and the other species is rare, thus indicating that “scarcity of conspecifics facilitates hybridization in general.” But in the same study Randler [14] found no evidence to suggest that females prefer mates of a larger species.

Haldane's [15] rule states that hybrid inviability occurs more frequently in the heterogametic sex. This phenomenon likely applies to all species that have sex chromosomes, and support for Haldane's rule has been demonstrated for F_1_ hybrid waterfowl. Kirby et al. [43] found evidence of post-mating isolation mechanisms in American black ducks (*Anas rubripes*) and mallards (*A. platyrhynchos*). Sixty-five percent of captive F_1_ hybrids were male, but the sex ratio did not differ for F_1_×F_1_ offspring or for F_1_ individuals backcrossed to parentals. Similar patterns are observed among domestic ducks [44]. Haldane's rule thus likely reduces the proportion of F_1_ female hybrids, but its effects may have only a minimal effect retarding interspecific gene flow, and identifying hybrid individuals other than F_1_ is generally not possible [45]. Tubaro and Lijtmaer [12] found that hybrid males outnumbered hybrid females, but this pattern was not found to be significant after correcting for male-biased sex ratios observed in adult duck populations.

The capacity for genes to introgress from one species to another has also been investigated in a variety of waterfowl species. Muñoz-Fuentes et al. [40] found that ruddy ducks (*Oxyura jamaicensis*) and white-headed ducks (*O. leucocephala*) hybridized frequently and produced viable offspring, but like our study in Argentina they did not find evidence of extensive introgression. On the other hand, Mank et al. [37] found evidence of extensive introgression between American black ducks and mallards. Peters et al. [38] likewise found an interesting example of ancient hybridization in the gadwall (*Anas strepera*), in which 5.5% of individuals sampled from North America possess heterospecific, falcated duck (*Anas falcata*) mtDNA haplotypes. Several other instances of mtDNA haplotype sharing have also been attributed to hybridization in waterfowl [24], [42], [46]. Such patterns are common in both mtDNA and nuclear DNA for a wide variety of other species in which hybridization has been examined using molecular methods [47], [48], [49], [50], [51], [52], [53].

In this regard our discovery of an F_1_ hybrid speckled teal x pintail and her duckling is not unusual. It is possible that it was just an isolated rare occurrence, but what is noteworthy is where it occurred, under what circumstances, and additionally where evidence of hybridization was not detected. Speckled teal and yellow-billed pintails are not sister taxa [54]. Nonetheless, they occur in widespread sympatry and are abundant thoughout their range in South America. Moreover, they frequently occur in mixed flocks, and to the casual observer, they are similar in appearance. Unlike any other waterfowl in South America, both species have yellow bills and brown plumage. In the Falkland Islands, however, yellow-billed pintails are outnumbered by speckled teal by approximately ten to one. It is not known whether yellow-billed pintails were formerly more abundant or if they always existed on the islands at low densities. Woods and Woods [19] reported that they have been uncommon since at least 1860. Only a couple pintails were observed in the Falkland Islands during our study period, and none were captured or banded. Nonetheless, we identified an F_1_ hybrid and her duckling with only a small sample of speckled teal (*n* = 15), compared to a much larger sample of both yellow-billed pintails and speckled teal (*n* = 120) in Argentina, in which no such hybrids were detected. Nor was firm evidence of introgression detected in Argentina with the three-population “isolation with migration” coalescent analysis. Our results thus provide further support for Hubbs' [16] “desperation hypothesis,” which states that scarcity in one population and abundance of another will often lead to hybridization. Our findings are also relevant to Haldane's [15] rule, because the F_1_ hybrid that was identified in this case was female (the heterogametic sex), and she successfully hatched a duckling. Based on her mtDNA she resulted from a pairing between a male yellow-billed pintail and female speckled teal. Yellow-billed pintails are among the two largest *Anas* dabbling duck species in South America, whereas speckled teal is the smallest. So although Randler [14] found no support for the hypothesis that females should prefer bigger males when mating heterospecifically, this was not necessarily the case in our study.

Finally, our study raises questions about small populations inhabiting oceanic islands. Island endemic populations are well known for their demographic properties and unique adaptations to insular environments [55], [56], [57], [58]. Island populations have additionally been shown to have lower genetic diversity, which may contribute to empirically higher rates of extinction [59], [60]. Speckled teal in the Falkland Islands were found to be significantly differentiated from populations in Argentina in their mtDNA and at three nuclear loci (Table 4, Fig. 3). Similar patterns of allelic endemism have recently been found among other island duck populations, including several species that have excellent dispersal capabilities and otherwise exhibit minimal geographic structure at continental scales: mallards [61], red-breasted mergansers (*Mergus serrator*; [62]), and green-winged teal (*Anas crecca*; J. Peters, pers. comm.). Speckled teal and yellow-billed pintails have excellent dispersal capabilities, and our study found that gene flow from Argentina to the Falkland Islands, in the direction of the strong prevailing winds in Patagonia [57], [63], is likely >100-fold greater than background rates of mutation. But given the small effective population size on the Falkland Islands, this may still equate to a small number of annual immigrants. Even if *M* were 1,000-fold greater than the mutation rate, the number of immigrants would be less than ten per generation. Gene flow in the opposite direction is likely occuring at substantially lower levels relative to mutation. Given that the number of effective immigrants is not high, it is thus no surprise that differention was found in mtDNA and other loci, particularly as female waterfowl have been repeatedly shown to exhibit high levels of philopatry and breeding site fidelity [64], [65]. In sum, speckled teal inhabiting the Falkland Islands likely comprise a distinct demographic unit, and while significant numbers of immigrants probably arrive from Argentina annually, gene flow is likely restricted and it may be that the Falkland Islands population is predominantly resident and non-migratory.

Similar information about yellow-billed pintails in the Falkland Islands is not yet available because of their scarcity. It is not known whether the species experienced a bottleneck prior to the first acounts in 1860 or always occurred at low densities. Regardless, the effect of hybridization on the yellow-billed pintail population would be of interest because of their small population size in the Falkland Islands. Hybridization has been shown to be an important factor leading to population declines [3], [66]. Its effects may be exacerbated in island ecosystems, particulary when one species is common but another is rare, and when pre- and post-zygotic barriers are porous or weakly developed [67]. Hybridization and backcrossing to speckled teal such as we observed here could potentially be one factor that has contributed to persistently low population numbers of yellow-billed pintails. More work in the Falkland Islands is clearly needed to answer these questions and determine the full extent of introgression.
